# Supplementary material for: Angelica sinensis polysaccharide as potential protectants against recurrent spontaneous abortion: focus on autophagy regulation
Source: Front Med (Lausanne). 2025 Jan 15;12:1522503. doi: 10.3389/fmed.2025.1522503 (PMC11774876; doi:10.3389/fmed.2025.1522503)
Supplement: Supplementary material S1 — The main instruments used during the LC-MS process, along with their models/specifications and manufacturers. [file Supplementary_file_1.zip › Metabolomics sequencing data FC1.2/6.差异代谢物/z-score-top20/Exposure_Ctrl.pdf]

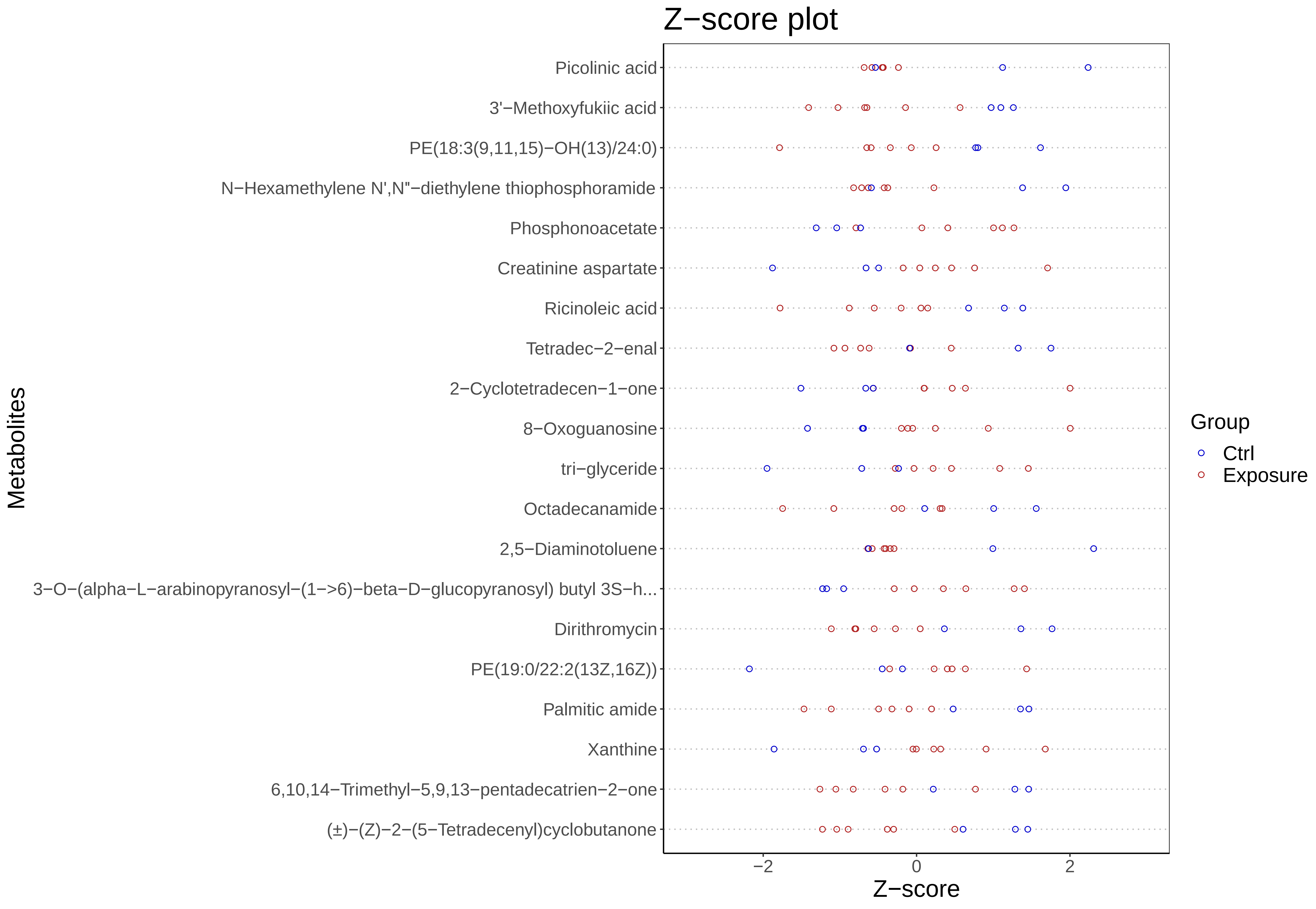
Z−score plot
Picolinic acid
3'−Methoxyfukiic acid
PE(18:3(9,11,15)−OH(13)/24:0)
N−Hexamethylene N',N"−diethylene thiophosphoramide
Phosphonoacetate
Creatinine aspartate
Ricinoleic acid
Tetradec−2−enal
2−Cyclotetradecen−1−one
8−Oxoguanosine
tri−glyceride
Octadecanamide
2,5−Diaminotoluene
3−O−(alpha−L−arabinopyranosyl−(1−>6)−beta−D−glucopyranosyl) butyl 3S−h...
Dirithromycin
PE(19:0/22:2(13Z,16Z))
Palmitic amide
Xanthine
6,10,14−Trimethyl−5,9,13−pentadecatrien−2−one
(±)−(Z)−2−(5−Tetradecenyl)cyclobutanone
Metabolites
−2
0
2
Z−score
Group
Ctrl
Exposure
